# Vascular endothelial growth factors C and D and lymphangiogenesis in gastrointestinal tract malignancy

**DOI:** 10.1038/sj.bjc.6601145

**Published:** 2003-07-29

**Authors:** S E Duff, C Li, M Jeziorska, S Kumar, M P Saunders, D Sherlock, S T O'Dwyer, G C Jayson

**Affiliations:** 1 Department of Surgery, Christie Hospital NHS Trust, Wilmslow Road, Manchester M20 4BX, UK; 2 Laboratory Medicine Academic Group, University of Manchester, Stopford Building, Oxford Road, Manchester M13 9PT, UK; 3 Department of Clinical Oncology, Christie Hospital NHS Trust, Wilmslow Road, Manchester M20 4BX, UK; 4 Department of Surgery, North Manchester General Hospital, Delauneys Road, Manchester M8 5RB, UK; 5 Department of Medical Oncology, Christie Hospital NHS Trust, Manchester M13 9PT, UK

**Keywords:** gastrointestinal neoplasms, lymphangiogenesis, lymphatic system, endothelial growth factors

## Abstract

Vascular endothelial growth factor-C (VEGF-C) and VEGF-D are members of the VEGF family of cytokines and have angiogenic and lymphangiogenic actions. In gastric adenocarcinoma, VEGF-C mRNA and tissue protein expression correlate with lymphatic invasion, lymph node metastasis and in some reports, venous invasion and reduced 5-year survival. Patients with gastric adenocarcinomas containing high levels of VEGF-C expression have significantly reduced 5-year survival rates, and VEGF-C expression is an independent prognostic risk factor for death. The role of VEGF-C in oesophageal squamous and colorectal cancer and VEGF-D in colorectal cancer is not clear, with conflicting reports in the published literature. In order to exploit potential therapeutic applications, further research is necessary to define the precise roles of these cytokines in health and disease.

Lymphangiogenesis, the development of new lymph vessels, is a relatively new area of clinical investigation. Increased interest in this field has been heightened by the discovery of new vascular endothelial growth factor (VEGF) family members, which possess lymphangiogenic roles.

Vascular endothelial growth factor-C (VEGF-C) and VEGF-D are secreted glycoproteins that are structurally similar, sharing areas of homology with one another and with the angiogenic growth factor VEGF-A (Joukov *et al*, 1996; Achen *et al*, 1998). They are specific ligands for the tyrosine kinase receptor, vascular endothelial growth factor receptor (VEGFR)-3 (flt-4) (Joukov *et al*, 1996; Achen *et al*, 1998). Both cytokines are subject to proteolytic processing, which also enables them to act as ligands for VEGFR2 (KDR/flk-1) (Joukov *et al*, 1997; Stacker *et al*, 1999). Vascular endothelial growth factor receptor 2 is expressed on vascular endothelial cells and is essential for the embryonic differentiation of endothelial and haematopoietic cells and formation of blood vessels (reviewed in Veikkola *et al*, 2000). Vascular endothelial growth factor receptor 3 is expressed on vascular endothelium early in development and on angiogenic endothelium, but is mainly restricted to the lymphatic endothelium in the adult (Kaipainen *et al*, 1995). Consequently, VEGF-C and D are implicated through their receptor affinities in angiogenic and lymphangiogenic pathways in health and disease (Stacker *et al*, 2002).

## ROLES OF VEGF-C AND VEGF-D

Study of the lymphatic system and lymphatic endothelial cells has been limited by a lack of specific lymphatic vessel markers, lack of lymphatic endothelial cells for culture and limited animal models. These problems are currently being overcome with a variety of methods. The recent discovery of specific lymphatic vessel markers, such as the hyaluronan receptor LYVE-1, podoplanin and Prox-1, new antibodies to these markers and antibody combinations has aided the identification of lymphatic vessels in histological specimens (Stacker *et al*, 2002) (Table 1

**Table 1:** Immunohistochemical staining methods for the detection of lymphatic endothelial cells

	**Sites of antigen expression**	**Advantages**	**Disadvantages**
*Single staining methods*			
VEGFR3 antibody	LEC, tumour neovasculature	LEC specific in normal tissue	Lack of specificity in tumours, VEGFR3 upregulated in angiogenic endothelium
Podoplanin antibody	LEC, glomerular podocytes	LEC specific	Lack of commercial antibody availability
Prox-1 antibody	LEC	LEC specific	Lack of commercial antibody availability
LYVE-1 antibody	LEC, hepatic sinusoidal cells	LEC specific	Lack of commercial antibody availability
Enzyme histochemistry (5′NA activity)	All endothelial cells at different levels		Findings difficult to interpret Hampered by nonspecific staining
			
*Double staining methods*			
CD31/PAL-E antibodies	Pan-EC marker/BEC	Enhanced discrimination accuracy	Frozen tissue required
VEGFR3/PAL-E antibodies	LEC, angiogenic BEC/BEC	Enhanced discrimination accuracy	Frozen tissue required
LYVE-1/CD34 antibodies	LEC/Pan-EC marker	Enhanced discrimination accuracy	Lack of commercial antibody availability
Prox-1/CD31 antibodies	LEC/Pan-EC marker	Enhanced discrimination accuracy	Lack of commercial antibody availability

LEC=lymphatic endothelial cells; 5′NA=5′nucleotidase; LYVE-1=lymphatic vessel endothelial hyaluronan receptor-1; BEC=blood vascular endothelial marker; Pan-EC marker=pan-endothelial cell marker.

). The exploitation of the differential expression of these new specific cell surface markers by lymphatic and blood vascular endothelial cells has allowed the separation of stable lymphatic cell populations for study (Podgrabinska *et al*, 2002). Animal models have been adapted from angiogenesis research and specific tumour, transgenic and knock-out models developed.

Our current understanding of the roles of VEGF-C and VEGF-D is derived mainly from *in vitro* and *in vivo* studies. *In vitro* studies have shown that VEGF-C and VEGF-D exhibit mitogenic effects for vascular and lymphatic endothelial cells and survival-promoting abilities for lymphatic endothelial cells through VEGFR3 (Joukov *et al*, 1997; Achen *et al*, 1998; Marconcini *et al*, 1999; Veikkola *et al*, 2001). Both growth factors promote angiogenesis in *in vitro* assays (Joukov *et al*, 1996; Joukov *et al*, 1997; Marconcini *et al*, 1999). Vascular endothelial growth factor-C promotes the formation of capillary-tube structures by lymphatic endothelial cells, but not blood vascular endothelial cells, in a collagen sandwich assay (Podgrabinska *et al*, 2002).

*In vivo* studies, using models adapted from angiogenesis research, have confirmed the angiogenic abilities of VEGF-C and VEGF-D and the lymphangiogenic effect of VEGF-C (Oh *et al*, 1997). Transgenic mouse models, which overexpress VEGF-C or VEGF-D in the epidermis, have shown cytokine-dependent, VEGFR3-mediated dermal lymphatic vessel enlargement and lymphatic endothelial cell proliferation without alteration in blood vasculature (Jeltsch *et al*, 1997; Makinen *et al*, 2001; Veikkola *et al*, 2001). Various tumour models have been constructed in which overexpression of VEGF-C or VEGF-D is demonstrated. These studies consistently show increased aggressiveness of the transfected cancer cell lines, intratumoural lymphangiogenesis, dilated and increased numbers of peritumoural lymphatics, enhanced rates of lymph node metastasis and increased tumour angiogenesis (Karpanen *et al*, 2001; Mandriota *et al*, 2001; Skobe *et al*, 2001).

Despite the implication of VEGF-C and VEGF-D in lymphangiogenic and angiogenic pathways in these studies, the role of the growth factors in the progression of human malignancy is unclear and the existence of functional lymphatics and lymphangiogenesis in human malignancy has been debated (Leu *et al*, 2000; Clarijs *et al*, 2001; Padera *et al*, 2002). Recent studies in head and neck cancer (Beasley *et al*, 2002; Maula *et al*, 2003) and melanoma (Straume *et al*, 2003) have demonstrated the existence of proliferating intratumoral lymphatic vessels. Further research is required to determine whether this is the case for all the different human malignancies that spread predominantly by the lymphatic route. The situation is likely to be clarified further by the use of antibodies and antibody combinations for the more specific lymphatic markers in conjunction with functional assays.

## VASCULAR ENDOTHELIAL GROWTH FACTOR-C AND VEGF-D IN HUMAN MALIGNANCIES

The dissemination of malignant cells to the regional lymph nodes is an early step in the progression of many common solid tumours and is an important determinant of prognosis. Positive associations have been found between the expression of VEGF-C in human malignant tissue with adverse clinicopathological features including lymphatic invasion and lymph node metastasis. Expression of VEGF-C mRNA is increased in a variety of human malignancies (Salven *et al*, 1998). Tumour types investigated include breast, gastric, colorectal, oesophageal, prostate, pancreas, cervical, thyroid, non-small-cell lung cancers, lung adenocarcinoma and laryngeal cancers. Clinically important areas of interest are the association between VEGF-C and -D expression, intra- and peritumoral lymphatic density, lymphatic and venous invasion, lymph node metastasis and survival.

### Methodological considerations

Many published reports conflict in their outcomes and conclusions. This may be partly explained by the use of different methodological tools and assumptions by their authors.

Immunohistochemical techniques and microvessel counting examine the tissue as near its condition *in vivo* as possible. Even so, results obtained examining malignant tissue at the invasive edge of tumours may not concur with results from central and superficial parts of the tumour (Furodoi *et al*, 2002). Scoring methods for both immunohistochemical staining and vessel counting vary between studies, with consequent difficulties in the extrapolation of results. Furthermore, the subjective nature of assessment of staining intensity and the frequent lack of positive or negative tissue controls in immunohistochemical analyses can confound analysis.

Studies examining mRNA levels provide an estimate of overall expression in the tissue fragment analysed, including tumour cells, stroma and normal mucosa, as RNA extraction necessarily entails tissue disruption. The nature of the interaction between expressed cytokines and the tumour microenvironment is at the cellular and paracrine level (Furodoi *et al*, 2002). Consequently, analysis of global tumour mRNA levels may miss subtleties of tissue expression that are crucial for tumour behaviour. The expression of mRNA in a tissue fragment may not necessarily equate with the expression of protein by the tumour.

Evidence for tumour-related lymphangiogenesis is derived from the presence of intratumoral lymphatics in xenograft studies. However, these vessels may be trapped in the tumour mass as a consequence of the methodology of model construction. Consequently, studies involving transgenic animals overexpressing VEGF-C, in which dilation of peritumoral lymphatics are seen (Mandriota *et al*, 2001) may reflect the situation in spontaneously arising human tumours more accurately (Karpanen and Alitalo, 2001).

Further discussion will focus on the current evidence for the role of VEGF-C and VEGF-D and their signalling receptors for the common sites of malignancy of the gastrointestinal tract (Table 2

**Table 2:** Immunohistochemical examination of VEGF-C expression in gastrointestinal malignancy

			**Clinicopathological associations of increased VEGF-C expression with regard to**				
**Tumour type**	**Number of cases**	**VEGF-C IHC positive (%)**	**Lymphatic invasion**	**Venous invasion**	**Lymph node metastasis**	**Prognosis**	**Reference**
Oesophageal SCC	48	40	***P*<0.01**	***P*<0.01**	***P*<0.01**	NA	Kitadai *et al* (2001)
Oesophageal SCC	71	54	*P*=0.51	*P*=0.092	*P*=0.085	*P*=0.80	Noguchi *et al* (2002)
Gastric adenocarcinoma	117	26	***P*<0.05**	***P*<0.01**	***P*<0.05**	***P*<0.001**	Yonemura *et al* (1999)
Gastric adenocarcinoma	76	45	***P*=0.04**	*P*=0.07	NS	NS trend	Ichikura *et al* (2001)
Early gastric adenocarcinoma	105	29	***P*=0.02**	NS increase	NS increase	NA	Kabashima *et al* (2001)
Gastric adenocarcinoma	65	51	***P*<0.05**	NS	***P*<0.05**	***P*<0.01**	Takahashi *et al* (2002)
Gastric adenocarcinoma	139	32	***P*<0.05**	NS	***P*<0.05**	NA	Amioka *et al* (2002)
Advanced colorectal adenocarcinoma	152	47	***P*<0.01**	***P*<0.01**	***P*<0.01**	***P*<0.05**	Furodoi *et al* (2002)
Colorectal adenocarcinoma	99	56	***P*<0.01**	NS	***P*<0.01**	NS trend	Akagi *et al* (2000)
Colorectal adenocarcinoma	59	35	NS	NA	NS	NA	George *et al* (2001)

SCC=squamous cell carcinoma; IHC=immunohistochemistry; NS=nonsignificant; NA=not assessed. Values in bold type indicate statistically significant results.

).

### Gastric cancer

Gastric cancer is a leading cause of cancer death worldwide. Lymph node status is important in the prediction of prognosis. Potential molecular markers that predict lymphatic involvement would improve the clinical management of this disease. The role of VEGF-C in predicting lymphatic invasion and lymph node metastasis in gastric cancer has been investigated in several studies (Table 2). There are no studies that have examined the role of VEGF-D in gastric cancer.

Immunohistochemical analysis of tumour tissue has demonstrated that VEGF-C immunoreactivity is restricted to gastric cancer cells and is observed diffusely throughout the cytoplasm (Yonemura *et al*, 1999, 2001; Ichikura *et al*, 2001). The percentage of gastric tumours that are positive for VEGF-C protein expression varies from 26 to 51% (Table 2) (Yonemura *et al*, 1999; Ichikura *et al*, 2001; Kabashima *et al*, 2001; Takahashi *et al*, 2002), although this may be accounted for in part by the use of varying methodology as discussed.

Lymphatic invasion and lymph node status correlate positively with tissue expression of VEGF-C in gastric cancer (Yonemura *et al*, 1999; Ichikura *et al*, 2001; Kabashima *et al*, 2001; Amioka *et al*, 2002; Takahashi *et al*, 2002) (Table 2). In addition, positive VEGF-C tissue expression in early gastric cancer (confined to the mucosa or submucosa) was significantly associated with lymphatic invasion, potentially helping to predict those individuals who would benefit from more or less extensive surgical resections (Kabashima *et al*, 2001). Similar associations have been demonstrated concerning the expression of VEGF-C mRNA expression in gastric cancer tissue. Malignant tissue expressed increased VEGF-C mRNA compared with adjacent normal mucosa (47 *vs* 13% (Yonemura *et al*, 1999); 55 *vs* 13% (Yonemura *et al*, 2001)). Furthermore, positive lymph node status, lymphatic and venous invasion were also associated with expression of VEGF-C mRNA (Yonemura *et al*, 1999).

The clinical impact of the association between VEGF-C expression and prognosis is not fully understood (Table 2). Nonsignificant trends towards reduced survival in VEGF-C expressing gastric cancers have been found (Ichikura *et al*, 2001). However, in 117 patients with gastric cancer, Yonemura *et al* (1999) demonstrated that high levels of VEGF-C expression were associated with poorer prognosis and decreased survival. Further significant differences in survival associated with VEGF-C status have been reported by Takahashi *et al* (2002) in a group of 65 cancer patients. A potentially important clinical finding of this study was the negative correlation of dendritic cell density with VEGF-C expression in the tumour. The effect of VEGF-C on survival may be due, in part, to its regulatory function on dendritic cells with potential reduced immunosurveillance of the tumour (Kabashima *et al*, 2001).

In contrast to VEGF-C, VEGFR3 immunoreactivity in gastric tumours is restricted to endothelial cells of mucosal and submucosal vessels that are regarded primarily as lymphatic vessels but also to a very few small blood vessels. Consequently, the majority of VEGFR3-positive vessels in gastric cancer are considered as lymphatics (Yonemura *et al*, 1999, 2001). A positive correlation between VEGFR3 and VEGF-C mRNA expression was seen in gastric cancer tissue specimens (Yonemura *et al*, 1999, 2001). Microvessel counts for VEGFR3 positive vessels showed a significant increase in VEGF-C mRNA positive tumours compared to VEGF-C mRNA negative tumours (6.96±6.05 *vs* 2.16±2.00, *P*<0.001). However, there was no overall increase in the VEGFR3 positive vessel count in tumour stroma compared with normal gastric mucosa when both VEGF-C mRNA positive and negative tumours were considered together (4.62±5.85 *vs* 2.48±1.64, *P*=0.067) (Yonemura *et al*, 2001). Similar increases in VEGFR3 positive vessel counts are seen in gastric cancers that are lymph node positive, show lymphatic invasion or are poorly differentiated (Yonemura *et al*, 2001).

In summary, in gastric cancer, expression of VEGF-C mRNA is higher in tumour than in normal mucosa. Vascular endothelial growth factor-C mRNA and immunohistochemically detected tissue expression of the protein in gastric cancer correlate with lymphatic invasion and lymph node metastasis and in some studies, venous invasion with reduced survival (Table 2). Vascular endothelial growth factor receptor 3 expression is mainly found on lymphatic vessels in gastric tumours and VEGFR3 mRNA levels and tissue expression parallel that of VEGF-C. These results suggest that VEGF-C and VEGFR3 act together in a paracrine fashion in the microenvironment of the gastric tumour.

### Oesophageal cancer

Oesophageal cancer has a poor prognosis, which is dependent on the presence of lymph node metastases. Limited and conflicting evidence exists for the role of VEGF-C in oesophageal cancer and no research is available concerning VEGF-D. Kitadai *et al* (2001) analysed the relationship between the expression of VEGF-C and clinicopathological characteristics in oesophageal squamous cell carcinoma. *In vitro* analysis demonstrated that four of the five oesophageal carcinoma cell lines studied expressed VEGF-C mRNA. *Ex vivo* analysis confirmed VEGF-C mRNA to be present in eight of the 12 oesophageal squamous carcinomas. In a further 48 archival specimens, 39.6% showed positive immunohistochemical staining for VEGF-C, which correlated with stage of disease, lymphatic invasion, venous invasion and lymph node metastasis (*P*<0.01) and depth of tumour invasion (Tumour *in situ* (Tis) *vs* T1, *P*<0.05; Tis *vs* T2, T3, *P*<0.01). Interestingly, the number of blood vessels detected by immunohistochemical staining for CD34 was significantly higher in the VEGF-C-positive tumours than the VEGF-C-negative tumours (Kitadai *et al*, 2001), suggesting that VEGF-C may be involved in both angiogenic and lymphangiogenic processes in tumours. However, a similar study examined larger numbers of oesophageal squamous carcinomas for immunohistochemical expression of VEGF-C protein, but did not report a significant association between the expression of the cytokine and any clinicopathological factor other than histological grade (Noguchi *et al*, 2002) (Table 2).

Vascular endothelial growth factor-C expression is associated with neoplastic progression in the oesophageal mucosa. Using immunohistochemical detection, normal oesophageal mucosa does not express VEGF-C although there is an increase in expression in Barrett's epithelium as it progresses through dysplasia to adenocarcinoma, and this is paralleled by a similar increase in VEGFR3 expression on lymphatic vessels (Auvinen *et al*, 2002).

### Colorectal cancer

Colorectal cancer is similar to oesophageal cancer, in that the role of VEGF-C is less well understood than in gastric carcinoma. Conflict also exists as to the role of VEGF-D. Recent publications illustrate conflicting results regarding protein and gene expression in relation to clinicopathological measures (Table 2).

With respect to VEGF-C expression, several authors have demonstrated associations between growth factor expression and poor clinicopathological outcome (Akagi *et al*, 2000; Furodoi *et al*, 2002). Immunohistochemical detection of VEGF-C expression at the deepest invasive site of colorectal carcinoma was found in 47% of 152 advanced tumours. Expression correlated with lymphatic and venous invasion, lymph node status, Dukes' stage, liver metastasis, depth of invasion, poorer histological grade and microvessel density (Furodoi *et al*, 2002). Vascular endothelial growth factor-C expression and lymph node metastasis were independent prognostic factors for 5-year survival on multivariate analysis (odds ratio (OR) 9.10, *P*=0.0272 and OR 8.52, *P*=0.0322, respectively). The study also emphasised the paracrine nature of the interaction between VEGF-C and the tumour microenvironment and the positive relationship between VEGF-C and tumour angiogenesis (Furodoi *et al*, 2002). Similar associations between tissue VEGF-C expression and clinicopathological factors have been described by Akagi *et al* (2000) with consistent patterns of VEGF-C expression in involved lymph nodes and primary tumours, although in this study only a nonsignificant trend towards decreased survival was identified in VEGF-C positive groups.

Contradictory evidence exists concerning the role of VEGF-C in lymphatic metastasis in colorectal cancer. Studies examining mRNA levels of various VEGF family members tend to show a lack of association with clinicopathological factors. George *et al* (2001) showed an increase in VEGF-A and VEGF-C mRNA in carcinomas (*P*=0.006 and *P*=0.004, respectively) but not in colonic polyps (*P*=0.22 and 0.5, respectively). No association was found between the increased level of VEGF-C mRNA and lymph node status, although a positive relationship existed between positive lymph nodes and VEGF-A mRNA expression. Patterns of VEGF-C mRNA expression were similar in the primary tumour and lymphatic metastases. The mRNA findings of the study were confirmed by immunohistochemistry, which showed no correlation between positive staining for VEGF-A, VEGF-C or VEGF-D and lymphatic spread (George *et al*, 2001). Further analyses of VEGF family mRNA levels in the adenoma–carcinoma sequence showed that of VEGF-A, VEGF-B and VEGF-C, only VEGF-A mRNA levels were consistently raised in invasive malignancy and this became apparent early on in disease progression, as levels were elevated to a similar extent in tumours with and without lymph node metastases or distant spread (Andre *et al*, 2000).

A few studies have focussed on the role of VEGF-D in colorectal malignancy with conflicting results. Tumour expression, assessed by RT-PCR, of VEGF-D mRNA was less than in normal tissue (George *et al*, 2001), while White *et al* (2002) found higher levels of VEGF-D protein expression in cancers detected by immunohistochemistry. The increased VEGF-D protein levels detected were associated with lymph node involvement and reduced overall and disease-free survival (White *et al*, 2002).

The role of VEGF-D within tumours is not well understood, but it has been suggested that VEGF-D may act competitively as an antagonist to the other VEGF family members. George *et al* (2002) postulated that a reduction in VEGF-D levels in the adenoma–carcinoma sequence allowed the more potent angiogenic cytokines VEGF-A and VEGF-C to bind more readily to the signalling receptors VEGFR2 and VEGFR3. The balance between various members of the VEGF family, their relative levels within a tumour, the extent of proteolytic processing and receptor availability may be important in determining tumour behaviour. The importance of the balance between VEGF-C and VEGF-D is illustrated in lung adenocarcinoma, where a low ratio of VEGF-D:VEGF-C (i.e., low VEGF-D and high VEGF-C) is associated with lymph node metastasis and lymphatic invasion (Niki *et al*, 2000).

Upregulation of cytoplasmic VEGFR3 protein expression has been demonstrated immunohistochemically in colorectal cancer tissue specimens and increased expression was associated with poorer overall survival (*P*<0.05) (Witte *et al*, 2002). This again demonstrates the potent paracrine nature of the interaction between the cytokines and their receptor in the microenvironment of the tumour.

In conclusion, conflicting reports exist for the precise involvement of VEGF-C and VEGF-D in lymphatic invasion, lymph node metastasis and prognosis in colorectal cancer. The importance of appropriate sampling and consistency in methodology of immunohistochemical staining and scoring are fundamental to interpretation and comparison between studies.

## CONCLUSIONS

Lymphangiogenesis is an exciting area of research in cancer biology. The growth factors VEGF-C and D are involved in this process and possess angiogenic and lymphangiogenic properties. The expression of lymphangiogenic factors is increased in many human malignancies and this is illustrated with respect to malignancies of the gastrointestinal tract. In gastric adenocarcinoma, lymphatic metastasis and lymphatic invasion are enhanced by increased expression of VEGF-C. The precise role for VEGF-C in colorectal and oesophageal squamous malignancy and VEGF-D in other tumours is not clearly understood, but is clearly important at a paracrine level. Further studies using combinations of new lymphatic markers and functional assays will help clarify the influence of these and other cytokines in the future. However, an essential requirement to allow comparison between studies is the development of consistent experimental methodology. This must include the use of antibodies of defined specificity, consistent immunohistochemical protocols with appropriate use of controls and widespread consensus in scoring techniques. Further understanding of the function and actions of VEGF-C and VEGF-D is required to optimise therapeutic strategies, avoiding unwanted side effects, in the treatment of benign and malignant disease.
